# A Flexible Terahertz Metamaterial Biosensor for Cancer Cell Growth and Migration Detection

**DOI:** 10.3390/mi13040631

**Published:** 2022-04-16

**Authors:** Weihao Fang, Xiaoqing Lv, Zhengtai Ma, Jian Liu, Weihua Pei, Zhaoxin Geng

**Affiliations:** 1 State Key Laboratory for Integrated Optoelectronics, Institute of Semiconductors, Chinese Academy of Sciences, Beijing 100083, China; fwh@semi.ac.cn (W.F.); lvxiaoqing284@semi.ac.cn (X.L.); mazhengtai@semi.ac.cn (Z.M.); peiwh@semi.ac.cn (W.P.); 2 College of Materials Science and Opto-Electronic Technology, University of Chinese Academy of Sciences, Beijing 100049, China; 3 Key Laboratory of Superlattices and Microstructures, Institute of Semiconductors, Chinese Academy of Sciences, Beijing 100083, China; liujian@semi.ac.cn; 4 School of Information Engineering, Minzu University of China, Beijing 100081, China

**Keywords:** terahertz metamaterial, biosensor, cell migration, transform growth factor-*β*

## Abstract

Metamaterial biosensors have been extensively used to identify cell types and detect concentrations of tumor biomarkers. However, the methods for in situ and non-destruction measurement of cell migration, which plays a key role in tumor progression and metastasis, are highly desirable. Therefore, a flexible terahertz metamaterial biosensor based on parylene C substrate was proposed for label-free and non-destructive detection of breast cancer cell growth and migration. The maximum resonance peak frequency shift achieved 183.2 GHz when breast cancer cell MDA−MB−231 was cultured onto the surface of the metamaterial biosensor for 72 h. A designed polydimethylsiloxane (PDMS) barrier sheet was applied to detect the cell growth rate which was quantified as 14.9 µm/h. The experimental peak shift expressed a linear relationship with the covered area and a quadratic relationship with the distance, which was consistent with simulation results. Additionally, the cell migration indicated that the transform growth factor-*β* (TGF-*β*) promoted the cancer cell migration. The terahertz metamaterial biosensor shows great potential for the investigation of cell biology in the future.

## 1. Introduction

Metamaterials are types of artificial media composed of periodically arranged subwavelength structures. They exhibit many unique properties that could not exist in natural materials and have attracted great attention recently [1,2,3,4,5,6]. Metamaterials especially generated resonant absorption peaks as a response to incident electromagnetic waves, which are sensitive to the change in the dielectric environment on the surface of metamaterials [7]. Moreover, THz radiation has quite low photon energy, effectively avoiding harmful ionization to biomolecules which determines their suitability for biosensing. Therefore, the metamaterial has been used in the sensing of protein [8], cell [9], and ribonucleic acid (RNA) [10]. Particularly, the effective sensing depth for the terahertz metamaterial biosensor is about 10 μm above the surface [11], which means metamaterials are more suitable for thick sample sensing, such as cell sensing. Several research papers about the detection of cells by terahertz metamaterial biosensors have been reported in recent years. For example, Zhang proposed a metamaterial biosensor with five concentric ring structures, which was used as a label-free and in situ detection tool in researching cell apoptosis [12]. The peak frequency shift depended on the change of the refractive index on the whole surface. Yang applied the metamaterial biosensor with an electromagnetically induced transparency (EIT) structure to detect different concentrations of lung cancer cells. In addition, the dielectric parameters of different cell concentrations were simulated to match experiment results [13]. Zhang distinguished different types of cells according to the resonance frequency shift and peak magnitude variation, which depended on the dielectric loss of the analyte [14]. Yan [15] applied EIT-type structural metamaterial biosensors to study the inhibitory effect of anti-cancer drugs on cancer cells. The current research mainly focuses on cell concentration detection and distinguishing the cell types [16], which are related to the change of dielectric parameters on the surface. However, research demonstrated that the resonance peak frequency of metamaterial biosensors is also sensitive to the cell distribution [17]. This special property indicates that the terahertz metamaterial biosensor could be applied to observe the dynamic processes of cells that depend on the cell position, such as cell growth and migration, which were never reported.

Cell migration plays a key role in various biological processes, such as angiogenesis, cancer metastasis, wound healing, and inflammation [18]. Traditional cell migration detection methods are the wound-healing assay [19] and the Boyden chamber assay [20], of which the processes are time-consuming and require professionals. The “wound” area is made by a pipette, which may damage the cells at the edge of the wound [21]. With the development of microfluidic technology, microfluidic-based wound-healing assays have been proposed [22,23,24]. This method still has some limitations, including complex manual operation [25], precise fluid control [26], and special materials [27]. Therefore, developing a simple, label-free, and non-destructive detection method for cell migration is highly required.

Here, we proposed a terahertz metamaterial biosensor based on a double U-shaped structure for label-free and non-destructive sensing of cells, offering a feasible strategy to study cell migration. The metamaterial structure was fabricated on a thin low-refractive-index flexible parylene substrate, which shows good performance in the detection of cell concentrations. A linear relation is found between cell area and the resonance peak frequency shift in simulation and experiment by introducing a polydimethylsiloxane (PDMS) barrier sheet as a “wound”, which means that our biosensor would quantify the cell growth. Moreover, the effects of transform growth factor-*β* (TGF-*β*), which could promote MDA−MB−231 cell migration [28], were investigated by the biosensor. The results indicate that cells migrate further when the concentration of TGF-*β* is increased, which is consistent with the biological method. This terahertz metamaterial biosensor exhibits great promise for future biological and biomedical detection of cancer cells.

## 2. Materials and Methods

### 2.1. Design and Fabrication of Metamaterial

**Figure 1:**
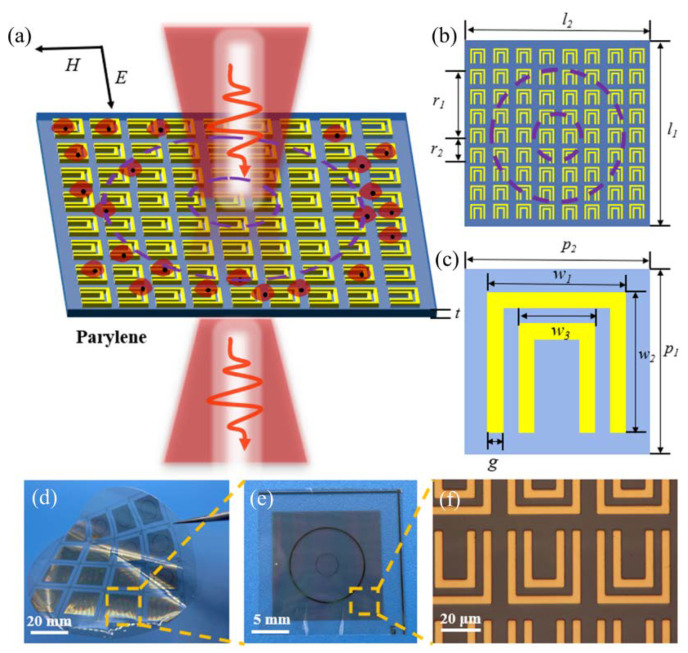
(**a**) Schematic illustration of the metamaterial biosensor: THz beams normally incident through the biosensor on which the MDA−MB−231 cell was cultured; (**b**) the structure of the metamaterial biosensor. The geometrical parameters are *l*_1_ = *l*_2_ = 20 mm, *r*_1_ = 5 mm, *r*_2_ = 1.5 mm, and *t* = 15 µm, respectively; (**c**) the structure of double U-shaped metamaterial. The geometrical parameters are *p*_1_ = *p*_2_ = 44 µm, *w*_1_ = *w*_2_ = 36 µm, *w*_3_ = 2 = 0 µm, and *g* = 4 µm, respectively; (**d**) physical photograph of 4-inch biosensor; (**e**) physical photograph of 20 × 20 mm biosensor; (**f**) micrograph of the metamaterial.

Figure 1a–c shows the experimental scheme and geometric parameter dimensions. The metamaterial is composed of periodic double U-shaped metal structures, which were fabricated on a 15 µm thick parylene C substrate. The dielectric constant of parylene C is 2.7, which is smaller than traditional substrate materials quartz and polyimide (PI). That means it is more suitable for sensing. The parylene C substrate was grown on a 4-inch silicon wafer by chemical vapor deposition (CVD) (PDS2010, Specialty Coating Systems company). Then the metamaterial structure was fabricated on the parylene C substrate by a standard UV lithography method. Next, 5 nm of Cr and 100 nm Au was deposited on the parylene C by electron beam evaporation. Then, the 4-inch biosensor was peeled off from the Si wafer in water, and the biosensor was cut into a 20 mm × 20 mm sensing unit. Figure 1d, e shows photographs of the 4-inch biosensor and the sensing unit. The radiuses of the two circles were *r*_1_ = 5 mm and *r*_2_ = 1.5 mm, respectively, which were used for locating the PDMS chamber and barrier sheet. Figure 1f is the microscope image of the double U-shaped metamaterial.

### 2.2. Cell Growth on Biosensor

**Figure 2:**
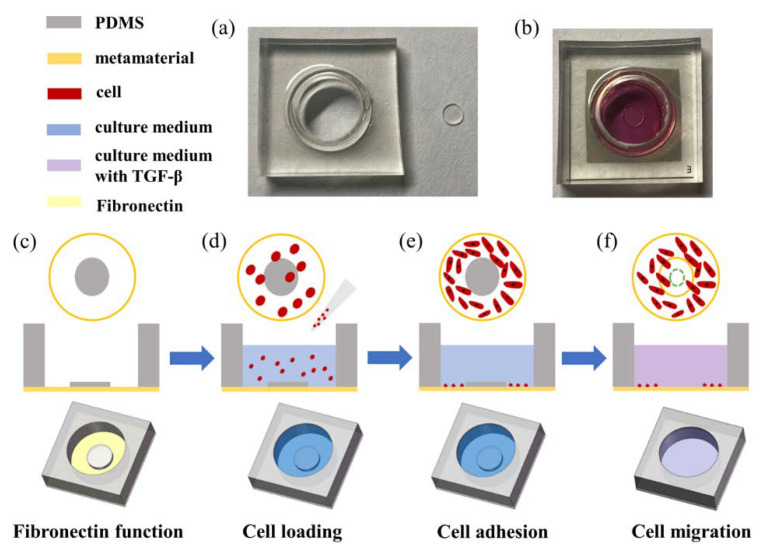
(**a**) Photograph of PDMS chamber and barrier sheet; (**b**) photograph of biosensor after cell loading; (**c**–**f**) schematic diagram of the process of cell migration experiment; (**c**) fibronectin function; (**d**) cell loading; (**e**) cell adhesion; (**f**) cell migration.

To quantify the growth and migration of cells, we proposed a “wound-healing” device composed of a PDMS (Sylgard 184, Dow Corning, Midland, MI, USA) culture chamber and a PDMS barrier sheet. The device was prepared as follows. First, Sylgard 184 PDMS base and curing agents (Sylgard 184, Dow Corning Corp.) were mixed in the ratio of 10:1 and were degassed. Then the mixture was poured into glass containers with thicknesses of 4 mm (chamber) and 0.5 mm (barrier sheet) respectively. Second, the containers were put in an oven at 70 °C for 2 h to make the PDMS completely cured. Finally, PDMS films were cut into chambers and barrier sheets by molds. The radius of the culture chamber is 5 mm, and the radius of the barrier sheets is 1.5 mm, as shown in Figure 2a. The surfaces of PDMS and parylene fit well without leakage since both are hydrophobic. Figure 2b is the photograph after cell loading.

The human breast cancer cell lines MDA−MB−231 transfected with red fluorescent protein gene (MDA−MB−231−RFP) were obtained from China Infrastructure of Cell Line Resource. These cell lines were cultured in high-glucose Dulbecco’s Modified Eagle Medium (DMEM; HyClone) supplemented with 10% fetal bovine serum (FBS; HyClone), penicillin (100 U/mL, Gibco), and streptomycin (100 μg/mL, Gibco) and maintained at 37 °C in a humidified incubator with 5% CO_2_.

The operation of the cell growth and migration experiment is shown in Figure 2c–f, which mainly involved four steps: fibronectin function on the chip surface, cell loading, cell adhesion, and cell migration. Firstly, all the devices (PDMS chamber, PDMS barrier sheet, biosensor) were sterilized under UV light for 30 min. Then, the PDMS chamber was tightly bonded to the metamaterial as the hole edges aligned with the outer circle (*r*_1_ = 5 mm) to form the culture chamber. The PDMS chamber was treated with fibronectin solution (Millipore) at a concentration of 50 μg/mL before cell loading. Then, the PDMS barrier sheet was pasted on the biosensor aligned with the inner circle (*r*_2_ = 1.5 mm), which was used as a “wound” to prevent cell adhesion inside the inner circle (Figure 2c). Secondly, breast cancer cell line MDA−MB−231 at a density of 1 × 10^5^ cell/mL, which was transfected with a red fluorescent protein, was introduced into the cell chamber (Figure 2d). Thirdly, the whole device was maintained at 37 °C in a humidified incubator with 5% CO_2_ for promoting cell attachment and spreading. The PDMS barrier sheet was removed after the outer circle metamaterial surface was completely covered with cells (Figure 2e). Finally, the cell grew in the chamber by adding different culture mediums for different experiments (Figure 2f). There is no need to introduce the PDMS barrier sheet for the cell concentration examination. The cells grow directly in the PDMS chamber to verify the growth ability of cells on the surface of the metamaterial. For the cell growth rate test, the culture medium was DMEM supplemented with 10% FBS, 100 U/mL penicillin, and 100 μg/mL streptomycin. The cells gradually grow inward through the inner circular boundary after removing the PDMS barrier sheet. The growth rate would be achieved by analyzing the growth distance and resonance peak shift (∆f) against time. For cell migration detection, the culture medium contained TGF-*β*. The effects of TGF-*β* on breast cancer cell migration would be obtained by analyzing the migration distance and resonance peak shift (∆*f*) against the concentrations and time.

### 2.3. Fluorescence and Terahertz Spectroscopy Measurement

Fluorescent pictures were captured to observe cell growth and migration. The cells were washed with PBS, fixed with 4% paraformaldehyde for 10 min at room temperature after growing under different conditions. Then, a microscope (Ti-s, Nikon, Tokyo, Japan) with a CCD camera (Ds-Ri1, Nikon, Japan) was used to record the fluorescent pictures at a magnification of 20 times.

The THz transmission spectrum of terahertz metamaterials was measured by a commercial terahertz time-domain spectroscopy test system (CIP-TDS, Daheng Optics). The test environment temperature was 23 ± 0.5 °C, and the relative humidity was less than 5%. The cells were washed with PBS, fixed with 4% paraformaldehyde for 10 min at room temperature after growing under different conditions. Then, the fixed cells were rinsed with deionized water and sucked out. A dust-free paper was used to dry the residual water on the surface quickly. Finally, time-domain spectroscopy of the biosensor was measured as $E_{1}\left(\omega \right)$ by CIP-TDS, and spectroscopy of air was measured as $E_{2}\left(\omega \right),$ the transmissivity can be obtained by:

(1) $$T\left(\omega \right)=E_{1}\left(\omega \right)/E_{2}\left(\omega \right)$$

## 3. Result and Discussion

### 3.1. Simulation of Metamaterial Biosensor

**Figure 3:**
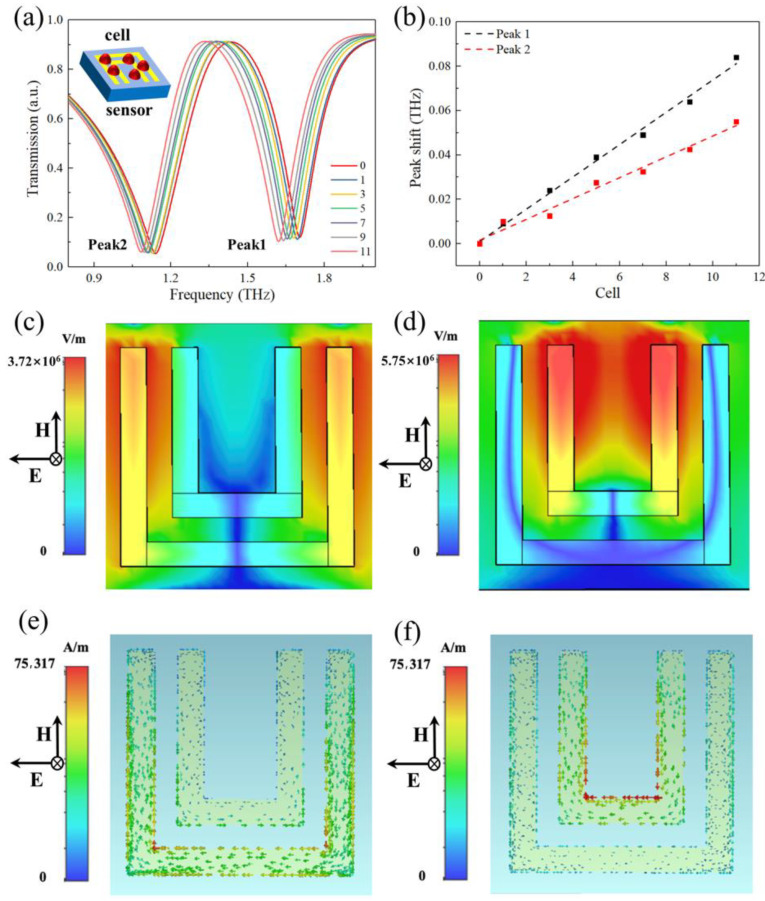
The simulated results of the metamaterial biosensor. (**a**) The transmission spectra of metamaterial with different numbers of cells on the surface. The inner graph shows the schematic illustration of the simulation; (**b**) the peak shift of the two peaks with different numbers of cells. (**c**) Electric field distribution in the *X-Y* plane at peak 2. (**d**) Electric field distribution in the *X-Y* plane at peak 1. (**e**) Surface current distribution in the *X-Y* plane at peak 2. (**f**) Surface current distribution in the *X-Y* plane at peak 1.

**Figure 4:**
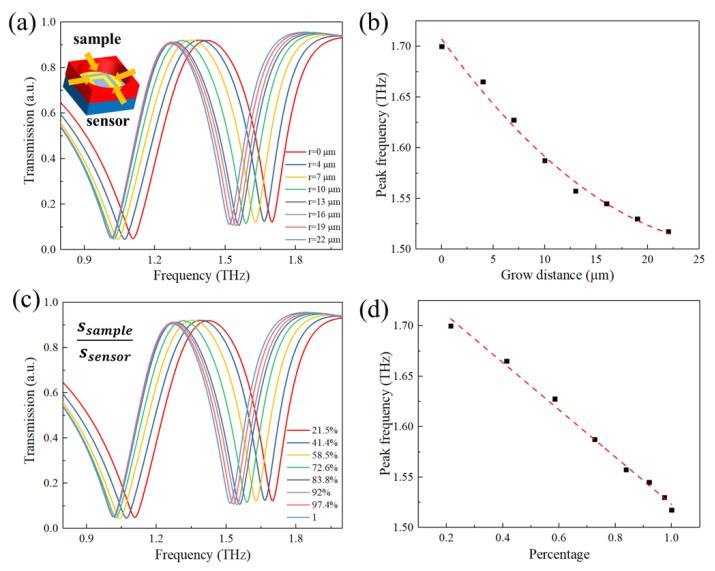
The cell growth simulations of the metamaterial biosensor. (**a**) The simulated transmission spectra of metamaterial under different cell growth distances. The inner graph shows the schematic illustration of simulation; (**b**) the dependence of peak 1 shift extracted from (**a**) on growth distance. (**c**) The simulated transmission spectra of metamaterial under different cell area ratios. (**d**) The dependence of peak 1 shift extracted from (**c**) on the growth distance area ratio of the cell.

The simulations of the double U-shaped metamaterial structure were completed by COMSOL Multiphysics. The parylene C and Au thickness were 15 μm and 100 nm, respectively. The dielectric constant of the parylene C substrate is *ε_p_* = 2.7. The conductivity of Au is *σ* = 4.56 × 10^7^ S/m, in accordance with the Debye distribution. The boundary conditions were periodic boundary conditions. The electric field of the incident electromagnetic wave was along the X direction, and the magnetic field was along the Y direction. To investigate the effect of the cell concentrations on the biosensor surface for resonance peak, different numbers of hemispheres, which were regarded as cells, were randomly put on the surface of the metamaterial. The radius of the hemispheres was 5 μm. The dielectric constant of the sample was 1.4. The transmittance simulation results are shown in Figure 3a. The insert figure was the schematic diagram of the simulation. The transmission curve had two absorption peaks when there were no cells on the surface of the metamaterial. The high-frequency peak (peak 1) was at 1.706 THz with a narrow half-width, and the low-frequency peak (peak 2) was at 1.12 THz with a wider half-width. The resonance peaks shifted to low frequency when the number of cells increased, indicating that the sensor could sense cell concentration on the surface. The slopes (shown in Figure 3b) indicated that the sensitivity of the high-frequency peak was higher than that of the low-frequency peak. Consequently, our follow-up research was focused on the high-frequency peak changes. Figure 3c, d exhibits the surface electric field distribution on the X-Y plane at the two resonance frequencies, respectively. The electric field is enhanced at the outer U ring for the low-frequency peak and at the inner U ring for the high-frequency peak. The surface current distributions in the X-Y plane at the two resonance frequencies are shown in Figure 3e, f. The results indicated that the low-frequency peak was formed by the LC oscillation of the outer U structure independently. On the other hand, the high-frequency peak is formed by the LC oscillation of the inner U structure. The values of capacitance and inductance are related to the size of the metamaterial. To investigate the dependence of the peak shift on the cell growth distance, a sample layer was set above the metamaterial as cells. The sample layer increased from the edge of the metamaterial unit to the center following a concentric circle. The thickness of the sample layer is 10 μm, and the refractive index is 1.4. The simulation result is shown in Figure 4a. The insert figure shows the simulation schematic diagram. The resonance peak frequency decreased as the growth distance increased. Figure 4b shows the relationship between the growth distance and the peak frequency. The fitting curve is a quadratic relation. According to the perturbation theory, the relative change of the resonant angular frequency $∆\omega /\omega_{0}$ is:

(2) $$\frac{∆\omega }{\omega_{0}}=\frac{-\int_{\nu_{0}}\left(∆\varepsilon {\left|{\bar{E}}_{0}\right|}^{2}+∆\mu {\left|{\bar{H}}_{0}\right|}^{2}\right)d\nu }{\int_{\nu_{0}}\left(\varepsilon {\left|{\bar{E}}_{0}\right|}^{2}+\mu {\left|{\bar{H}}_{0}\right|}^{2}\right)d\nu }\approx \frac{-\int_{\nu_{0}}\left(∆\varepsilon {\left|{\bar{E}}_{0}\right|}^{2}\right)d\nu }{2\int_{\nu_{0}}\left(\varepsilon {\left|{\bar{E}}_{0}\right|}^{2}\right)d\nu }$$

where *E*_0_ and *H*_0_ are the electric and magnetic fields of the environment above metamaterial, ∆*ε* is a change of the dielectric constant in the environment, and $\nu_{0}\;$is the effective integral volume. In our model, the thickness of the sample layer was 10 μm, which means the effective integral volume $\nu_{0}\;$was proportional to the area of the sample layer. Dielectric constant *ε* was set as 1.4, which would not change. Therefore, peak shift ∆*f* was proportional to the area of the sample layer. Figure 4a would be changed into Figure 4c when we calculated the ratio of *S_sample_*/*S_metamaterial_*. Figure 4d shows the relationship between the area ratios and peak frequency. The fitting equation was *y* = 1.7573 – 0.23*x*. The peak frequency was proportional to the area ratios, which was consistent with the theory. 

### 3.2. Cell Concentration Measurement

**Figure 5:**
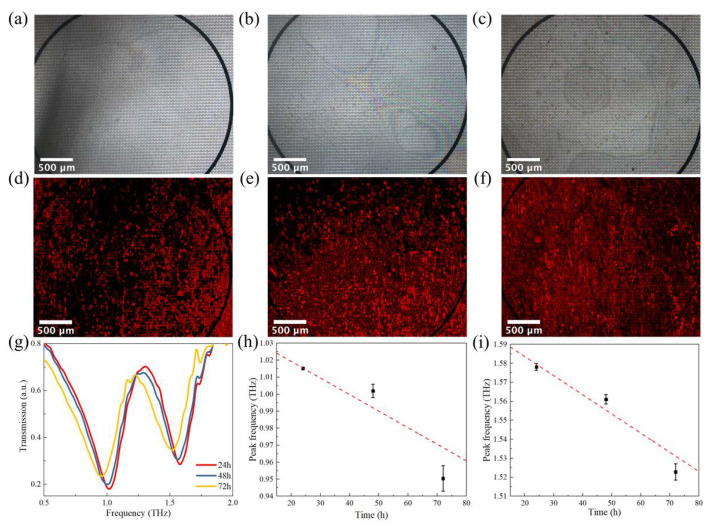
Experiment results of cells growing on the biosensor. (**a**–**c**) Micrograph of the biosensor with cells growing after 24 h, 48 h, and 72 h; (**d**–**f**) fluorescence pictures corresponding; (**g**) the transmission spectra of the biosensor with cells growing after 24 h, 48 h, and 72 h; (**h**) the dependence of peak 2 shift extracted from (**g**) on growth time. (**i**) The dependence of peak 1 shift extracted from (**g**) on growth time.

To investigate the sensing performance of the metamaterial biosensor, the breast cancer cells MDA−MB−231−RFP were directly cultured onto the biosensor’s surface without the PDMS barrier. Figure 5a–c shows the brightfield images of cells at 24, 48, and 72 h. The black border in the images was the boundary of the inner circle. Figure 5d–f shows the fluorescent images of cells at 24, 48, and 72 h. It could be found that the density of cells increased with time. The surface of the biosensor was almost completely covered after 72 h. The results showed that the cells grew and proliferated with good viability on the surface of the biosensor. Terahertz time-domain spectroscopy is shown in Figure 5g. The transmittance curves of cells gradually shift to the left as the growth time increases. Figure 5h,i shows the peak frequency versus grow time of the two resonance peaks, respectively. The slope of peak 1 was larger than peak 2, which indicated that the sensitivity of peak 1 was higher than peak 2. The experimental results are consistent with the simulation. The largest frequency shift of peak 1 was discovered as 183.2 GHz when the cells were cultured for 72 h. The experiment results illustrate that the THz metamaterial biosensor would be applied to detect the cell concentration.

### 3.3. Cell Growth Rate Quantification

**Figure 6:**
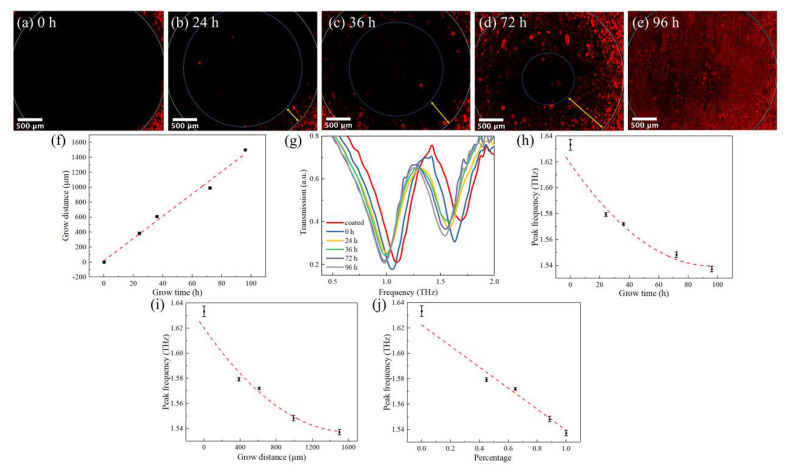
The cell growing behavior on the metamaterial biosensor. (**a**–**e**) Fluorescence pictures of the biosensor removing the PDMS barrier sheet for 0, 24, 36, 72, and 96 h. (**f**) The dependence of growth distance on time. (**g**) The transmission spectra of biosensor removing the PDMS barrier sheet for 0, 24, 36, 72, and 96 h. (**h**) The dependence of peak frequency extracted from (**g**) on time. (**i**) The dependence of peak frequency on growth distance. (**j**) The dependence of peak frequency on area ratios of cells and the inner circle.

The growth rate of cells was investigated by introducing the PDMS barrier sheet as “would”, which was used to prevent cells proliferating into the inner circle. As described in Figure 2e, f, the PDMS barrier sheet was removed when the cells completely covered the biosensor. Afterward, DMEM was put into the culture chamber to promote cell spreading. Figure 6a–e shows the fluorescent images of the cells at 0, 24, 36, 72, and 96 h. It could be clearly seen that cells gradually grew from the boundary of the inner circle to the center, eventually covering the whole surface of the biosensor at 96 h. The growth distance was measured by the microscope. Figure 6f shows the relationship between growth distance and time. The linear fitting function was *y* = 17.67 + 14.9*x*, indicating that the growth rate was 14.9 µm/h. The biosensors were measured by a time-domain spectroscopy system. The transmission curve is shown in Figure 6g. The curve shifted to the left as the growth time increases. The dependence of peak frequency on distance is shown in Figure 6h, the peak frequency and growth time have a quadratic relationship, of which the fitting function was *y* = 1.6186 – 0.0016*x* + 8.4 × 10^−6^*x*^2^. As the growth time increased, the peak frequency change gradually slowed down. The results are consistent with the simulation shown in Figure 4b. Figure 6i shows the peak position versus the growth distance, which is also a quadratic relationship. For further research on the relationship between cell growth and peak shift, the area ratio $S_{cell}/S_{circle}$ was calculated, where $S_{cell}$ was the area of cells entering the inner circle and $S_{circle}$ was the area of the inner circle (7.065 mm^2^). As shown in Figure 6j, the peak frequency was proportional to the area ratio, which was consistent with the simulation results. It proves that the terahertz metamaterial biosensor could quantify the growth rate of cells. Furthermore, the metamaterial biosensor could be applied to distinguish the cell types by the growth rate.

### 3.4. Effects of Transforming Growth Factors on Cell Migration

**Figure 7:**
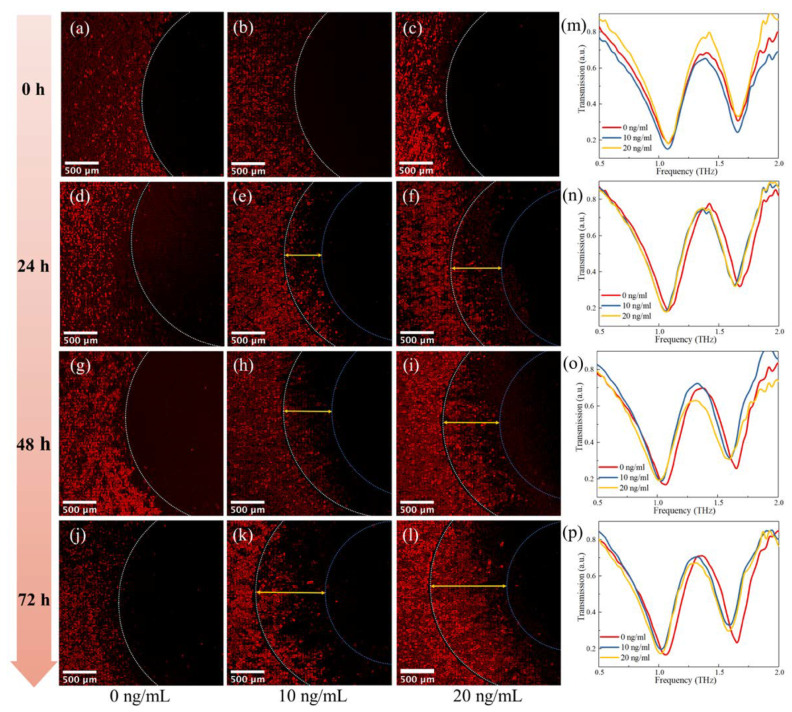
The cell migration behavior of MDA−MB−231 in response to different concentrations of TGF-*β*. (**a**–**l**) Fluorescence pictures of the biosensor under different concentrations of TGF-*β* for 0, 24, 48, and 72 h. (**m**–**p**) The transmission spectra of biosensor under TGF-*β* for 0, 24, 48, and 72 h.

**Figure 8:**
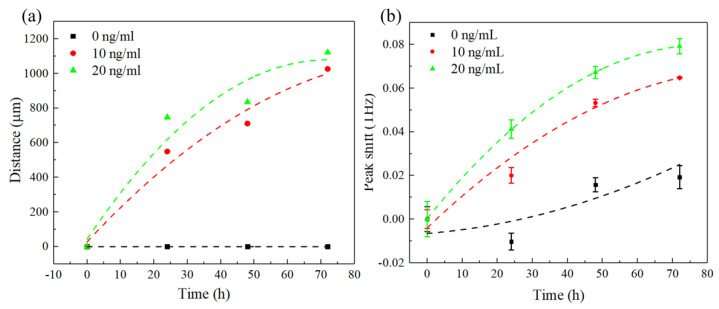
(**a**) The dependence of growth distance on time under different concentrations of TGF-*β* (0, 10, 20 ng/mL); (**b**) the dependence of peak shift on time under different concentrations of TGF-*β* (0, 10, 20 ng/mL).

Cell migration provides important information in various biological processes. Studies have demonstrated that TGF-*β* could promote MDA−MB−231 cell migration [29]. To expand the application of the biosensor, the effect of TGF-*β* on MDA−MB−231 cells migration was investigated without any cell damage by the biosensor. As shown in Figure 2f, the cells were incubated with the medium containing different concentrations of TGF-*β* (0, 10, 20 ng/mL) for different times (0, 24, 48, 72 h) after removing the PDMS barrier sheet. The biosensors were then recorded for fluorescent images and terahertz time-domain spectroscopy. Figure 7a–l shows fluorescent images of biosensors for varied conditions. It could be seen from Figure 7a–c that the cells were at the boundary of the inner circle at 0 h indicating that cells almost would not migrate without TGF-*β*. As shown in Figure 7d–l, the migration of MDA−MB−231 cells was enhanced as the concentrations of TGF-*β* increased at the same growing time. In addition, cell migration exhibited a positive correlation with time. The biosensors were measured by CIP−TDS and shown in Figure 7m–p. Obviously, the peak frequency shift to the left as the concentration of TGF-*β* increases from 0 ng/mL to 20 ng/mL. Figure 8a shows the growth distance related to the time. Cells move further when either time or concentration of TGF-*β* increases. The corresponding shift values of four growing times under different concentrations were extracted to acquire the dependence of frequency shift ∆f on the growing time. The peak shift $∆f^{j}$ at the TGF-*β* concentration of *j* ng/mL (j =0, 10, 20) is defined as $∆f^{j}=f_{i}^{j}-f_{0}^{j}$, where $f_{i}^{j}$ was the peak frequency of biosensor cultured for *i* h (*i* = 0, 24, 48, 72) at the concentration of *j* ng/mL, $f_{0}^{j}\;$was the peak frequency of biosensor cultured for 0 h at the concentration of *j* ng/mL. As shown in Figure 8b, the frequency shift increased as the time and concentration of TGF-*β* increased. After being cultured for 72 h, the peak frequency shift increased from 19.3 to 79.3 GHz when the concentration of TGF-*β* changed from 0 to 20 ng/mL. The spectrum results were consistent with the fluorescent results. That means that the migration of MDA−MB−231was significantly enhanced after adding TGF-*β*, which could be examined by our metamaterials without damage. The results of cell growth and migration experiments show that our biosensor was sensitive to the concentrations and the distributions of the cell on the surface of the metamaterial. The metamaterial was easily fabricated and measured. It could simply and quickly obtain the information of the cell without labeling. Moreover, the measurement of our biosensor was undamaged as the photon energy of the THz radiation was low.

## 4. Conclusions

A flexible terahertz metamaterial biosensor was proposed and fabricated, which was utilized for label-free non-destructive detection of cell growth and migration. The biosensor monitored the growth behavior of breast cancer cell MDA−MB−231 by introducing a PDMS barrier sheet as a “wound”. The linear relation between peak frequency shift and the area of the cell, which was explored through the simulation, has been confirmed experimentally. Furthermore, the effects of TGF-*β* on cell migration were investigated on the surface of the metamaterial biosensor. The measured results demonstrated that the migration of MDA−MB−231 would be significantly enhanced when increasing the concentration of TGF-*β*, which would cause a further peak shift. Therefore, the metamaterial biosensor offers a novel method for the detection of cell growth and migration and shows significant potential in the future biological and biomedical study of cancer cells.

## Data Availability

The study did not report any data.
